# Mediating role of psychological distress and alcohol use in socioeconomic disparities in deaths of despair: a causal mediation analysis using record linkage data

**DOI:** 10.1136/jech-2025-224372

**Published:** 2025-10-09

**Authors:** Aurélie M Lasserre, Yachen Zhu, Carolin Kilian, Laura Llamosas-Falcón, Jurgen Rehm, Charlotte Probst

**Affiliations:** 1 Addiction Medicine, Lausanne University Hospital Department of Psychiatry, Lausanne, Switzerland; 2 Institute for Mental Health Policy Research, Centre for Addiction and Mental Health, Toronto, Ontario, Canada; 3 Alcohol Research Group, Emeryville, California, USA; 4 National Institute of Public Health, University of Southern Denmark, Odense, Region Syddanmark, Denmark; 5 Danish Institute for Advanced Study, University of Southern Denmark, Odense, Region Syddanmark, Denmark; 6 Center for Interdisciplinary Addiction Research (ZIS), Department of Psychiatry and Psychotherapy, University Medical Center Hamburg-Eppendorf, Hamburg, HH, Germany; 7 Institute of Medical Science, University of Toronto Faculty of Medicine, Toronto, Ontario, Canada; 8 University of Toronto Dalla Lana School of Public Health, Toronto, Ontario, Canada; 9 Program on Substance Abuse & WHO European Region Collaboration Centre, Public Health Agency of Catalonia, Barcelona, Spain; 10 Campbell Family Mental Health Research Institute, Centre for Addiction and Mental Health, Toronto, Ontario, Canada; 11 Department of Psychiatry, University of Toronto Faculty of Medicine, Toronto, Ontario, Canada

**Keywords:** MENTAL HEALTH, MORTALITY, ALCOHOLISM, Health inequalities, RECORD LINKAGE

## Abstract

**Background:**

Deaths of despair – suicide, drug overdose and chronic liver disease mortality – are major contributors to premature mortality in the USA, disproportionately affecting individuals with low socioeconomic status (SES). The mechanisms underlying these disparities, particularly the roles of psychological distress and alcohol use, remain insufficiently understood. We assessed associations of SES, alcohol use and psychological distress with deaths of despair, along with the mediating roles of alcohol use and psychological distress in the SES-deaths of despair association in men and women.

**Methods:**

We linked US National Health Interview Survey data (1997–2018) to mortality records until 31 December 2019 by following 3 11 508 women and 2 42 463 men for 10.5 years. Using counterfactual-based inverse probability-weighted marginal structural models, we decomposed the total effect of SES (education, income) into direct and indirect effects through psychological distress (Kessler-6) and alcohol use (daily consumption). Analyses were sex-stratified and adjusted for marital status, race and ethnicity and survey year.

**Results:**

Severe psychological distress and high alcohol use were each associated with over a threefold increased risk of death of despair. In men, psychological distress and alcohol use mediated up to 16% and 14% of the SES-deaths of despair relationship, respectively. In women, psychological distress mediated up to 20% of the association, while alcohol use did not mediate the relationship.

**Conclusion:**

Low SES, psychological distress and alcohol use are key risk factors for deaths of despair. Intervention targeting mental health and alcohol use, especially through SES-specific and sex-specific approaches, may help reduce inequalities in these preventable causes of death.

**WHAT IS ALREADY KNOWN ON THIS TOPIC**

- Low socioeconomic status (SES) is strongly associated with higher mortality from deaths of despair (suicide, drug overdose and chronic liver disease).
- Alcohol is a leading cause of deaths of despair and is closely related to depression, increases the risk of suicide and drug and alcohol-related mortality.
- Research has mainly focused on the social determinants of deaths of despair, but knowledge on individual mental health, such as psychological distress, remains scarce.
- The interplays of SES, alcohol use and mental health in the deaths of despair remain unclear.

**WHAT THIS STUDY ADDS**

- Severe psychological distress increases the risk of death of despair by more than three times, at similar risk levels as high-level alcohol use (>60 g/day for men and >20 g/day for women).
- Higher levels of psychological distress and heavy alcohol use among lower SES groups increase the risk of deaths of despair in lower SES compared with higher SES.
- There is a clear difference between men and women regarding the effect of SES on deaths of despair. While psychological distress mediates the effect of SES on deaths of despair in both sexes, alcohol use is a key mediator only in men.

**HOW THIS STUDY MIGHT AFFECT RESEARCH, PRACTICE OR POLICY**

- Psychological distress and alcohol use should be key prevention targets to reduce deaths of despair, particularly in people with low SES.
- The causal pathway between SES and deaths of despair differs according to sex, suggesting sex-specific interventions to curb the deaths of despair epidemic.

## Introduction

 Life expectancy is shaped by the social conditions in which people live, including education, employment, housing, healthcare and social environment. Globally, health outcomes follow a clear social gradient: individuals with lower socioeconomic status (SES) tend to have worse health and higher mortality.^1 2^ This gradient is particularly pronounced in the USA, where life expectancy has decreased among two-thirds of the adults without a Bachelor’s degree (BA) since the 1990s, reversing decades of progress.^3^ In their landmark book ‘Deaths of Despair and the Future of Capitalism’ (2020),^4^ Case and Deaton described the profound social, economic and psychological distress, which they termed despair, experienced by individuals without a BA over recent decades. This despair stemmed from broad structural changes, including deindustrialisation, a weak social safety net and rising income inequality, further compounded by the weakening of community institutions, social ties and family cohesion.^3 4^ Three causes of death were mainly driving this increase in mortality: suicide, drug overdose and chronic liver disease, which they named deaths of despair because they involved a self-inflicted aspect through alcohol or drug use and, in extreme cases, suicide.^3 4^

The role of despair as the driving factor for deaths of despair has been the subject of debate. Other contributing factors were pointed out, such as the increasing affordability of alcohol and availability of opioids during the last decades.^5 6^ It was also argued that the term ‘despair’ lacked a clinical or theoretical definition.^7^ Moreover, the proposed causal pathway from socioeconomic conditions to despair and subsequently to deaths of despair mortality has not been formally tested.^7 8^ It is well demonstrated that individuals with lower SES often experience higher levels of psychological distress, such as anxiety or depressive symptoms,^9^ which are known risk factors for suicide^10^ and substance use disorders.^11 12^ Alcohol and drug use disorders are also closely related to depression,^12 13^ increasing the risk of suicide^10 14^ and alcohol or drug-related mortality.^10 13 15^ Thus, alcohol use and psychological distress might play a critical role in the pathways between SES and deaths of despair, but this has not been examined.

Men and women differ in patterns of substance use and psychological distress.^13 16^ Men are more likely to engage in heavy drinking and substance use. Women report higher levels of psychological distress and attempt suicide more often, though men have higher suicide mortality.^13 16^ Biological differences, as well as gendered roles, norms and stressors, shape these differences, necessitating separate analyses.^16 17^

The present study aimed (1) to estimate the association between SES, alcohol use and psychological distress with the risk of deaths of despair (suicide, drug overdose and chronic liver disease mortality) and (2) to examine the potential mediating effect of alcohol use and psychological distress in the association between SES and deaths of despair, in men and women separately.

## Methods

### Data source

Data from the annual, cross-sectional US National Health Interview Survey (NHIS) from 1997 to 2018 were linked to the 2019 National Death Index (NDI) using deterministic and probabilistic approaches.^18^ The NHIS provides nationally representative information on the health of the civilian, non-institutionalised US population residing in the 50 states and the District of Columbia. Of the total adult sample in NHIS 1997–2018 who had sufficient identifying data, 93% were eligible for mortality follow-up and were included in the present study. Every year, about 35 000 households are enrolled, from which one adult is randomly sampled for a face-to-face interview.^19^ NHIS is based on a multistage probability sampling design. Certain population groups, such as Hispanics, non-Hispanic Blacks and non-Hispanic Asians, are oversampled.^20^ Since the assessment of alcohol use was conducted in sufficient detail starting in 1997, NHIS data were pooled from 1997 to 2018. Follow-up on mortality status, underlying cause of death, time of death or last presumed alive, was available up to 31 December 2019 in the NDI.^21^ Participants younger than 25 years at the time of NHIS administration were removed on the assumption that they had not yet reached their final educational attainment (our primary SES indicator).

### Measures

All explanatory variables were obtained through face-to-face interviews at one time during the NHIS, whereas the causes of death were obtained from the NDI. The statistical outcome was the time between participation in the NHIS and the death of despair. Age was used as the time scale in survival analysis. The baseline age was calculated as the difference between the interview date and the participant’s date of birth. The end age was calculated as the difference between the date of death and the date of birth if the participant was deceased by 31 December 2019, and as the difference between 31 December 2019 and the date of birth otherwise. Following Case and Deaton’s definition, deaths of despair included the following causes of death according to the International Classification of Diseases, 10^th^ revision (ICD-10) codes: intentional self-harm (X60-X84 and Y87.0), drug (including alcohol) poisoning (X40-X45, Y10-Y14, Y45, Y47 and Y49) and chronic liver diseases (K70, K73 and K74).^4^ The latter includes alcoholic liver disease (K70), as well as other chronic liver diseases that can also be affected by alcohol use, by affecting the spontaneous clearance of viral infections, or by interacting with other risk factors for liver cirrhosis, for example.^22^ Sex was defined based on administrative records, referring to sex assigned at birth. The exposure of interest (SES) was primarily operationalised as educational attainment, given its critical role in disparities in mortality and life expectancy as described by Case and Deaton,^3^ and categorised as low (high school diploma or less, (reference)), medium (some college but no bachelor’s degree) or high (bachelor’s degree or more). In sensitivity analyses, SES was operationalised as income level using self-reported family income as a measure of income and categorised as high (>=400% of poverty threshold (reference)), medium (200%–399% of poverty threshold), low (<199% of poverty threshold) or missing.^23^

Alcohol use was categorised based on the mean grams of pure alcohol consumed per day according to the standards of WHO:^24^ (1) lifetime abstainers (never drank alcohol in the past 12 months and never had 12+drinks in any 1 year), (2) former drinkers (never drank alcohol in the past 12 months but had 12+drinks in any 1 year) and current drinkers who were categorised according to their past year daily average consumption into (3) category I (>0–20 g/d (men and women)), (4) category II (>20 g/d (women) or >20–40 g/d (men)), (5) category III (>40–60 g/d (men only)) and (6) category IV (>60 g/day (men only)). The latter two categories were for men only, as relatively few women consumed >40 g/day. Psychological distress was assessed using the validated Kessler-6 scale, which measures six symptoms (feeling ‘nervous’, ‘hopeless’, ‘restless or fidgety’, ‘so depressed that nothing could cheer (them) up’, ‘that everything was an effort’ and ‘worthless’) experienced in the past month, scored from 0 (none) to 4 (all the time). Total scores were classified as no/low (0–4), moderate (5-12) or severe (13-24) distress.^25 26^ Covariates included age, marital status (married or living with a partner vs never married, widowed, divorced or separated), race and ethnicity (non-Hispanic Black, Hispanic, others (including non-Hispanic Asian and Pacific Islander, American Indian and Alaska Native and non-Hispanic all other race groups) vs non-Hispanic White) and survey year.

### Statistical analysis

**Figure 1:**
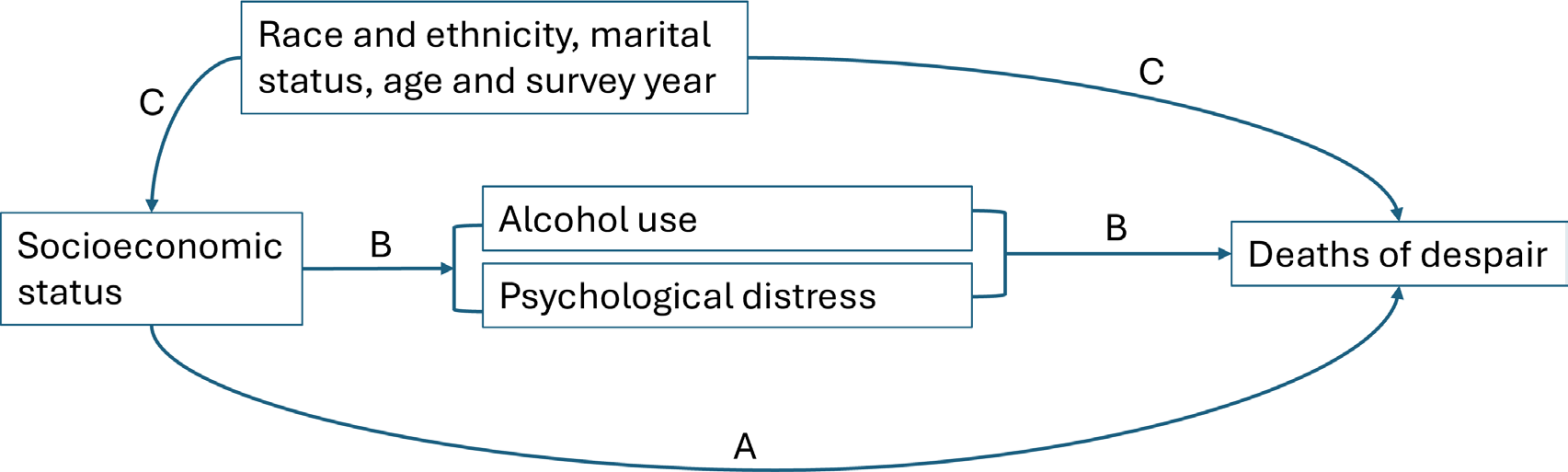
Schematic diagram of the association between socioeconomic status (SES) and deaths of despair mortality, with mediators and covariates. A: natural direct effect of SES on deaths of despair. B: natural indirect effect of SES on deaths of despair, mediated by alcohol use or psychological distress. C: confounding effect.

The complex survey design of the NHIS was accounted for by using survey weights, strata and primary sampling units using the survey package in R. Time-to-event analyses were first performed with traditional Cox proportional-hazards regression models to estimate the effects of SES, psychological distress and alcohol use on deaths of despair mortality, with age as the time scale. Using age (continuous) as the time scale allows for a complete non-parametric age effect, which provides more accurate risk relationships than using time-on-study as the time scale.^27^ Participants were censored at the date of death from causes other than deaths of despair or, if still alive, on 31 December 2019. Then, the association between SES and deaths of despair was decomposed into a mean natural direct effect and natural indirect effects through alcohol use and psychological distress, using causal mediation analyses based on inverse probability-weighted marginal structural models (figure 1),^28^ which provided valid estimates even in the case of non-collapsibility.^29^ While causal mediation analysis can provide insights into potential mechanisms (referred to as causal pathways), it does not support definitive conclusions about causality, particularly when residual confounding cannot be fully addressed. The calculation of mediation weight was explained in an earlier publication.^30^ The multiplication of the sampling weights and the mediation weights was incorporated into the model.^31^ Analyses were stratified by sex and adjusted for marital status, race and ethnicity and survey year.

Sensitivity analyses were conducted: (1) Using marginal structural models, the association between SES and death of despair mortality was decomposed into three components: the mean natural direct effect, the mean natural indirect effect through each mediator (differential exposure) and the mean effect of the mediated interaction between SES and each mediator (differential vulnerability) (online supplemental figure S1). (2) All models were repeated as sensitivity analyses, operationalising SES as income level.^23^ Due to the very few missing data (<5% in each variable),^30^ listwise deletion was applied, and complete case analysis was performed, assuming that the data are missing at random. Statistical analyses were conducted in R, V. 4.2.3.^32^

## Results

Online supplemental table S1 presents the weighted characteristics of the 311 508 female and 242 463 male participants by educational levels, respectively. The mean age of the participants was 50.2 years (SD=16.1), and the mean follow-up period was 10.5 years (SD=6.2). The prevalence of abstinence (lifetime abstinence or former drinking) *decreased* with the educational levels in men (37.0% in low to 21.1% in high educational level) and women (55.0% to 26.7%, respectively). In men, the prevalence of categories I and II drinking *increased* with educational levels (57.5% to 76.2%, respectively); conversely, categories III and IV drinking *decreased* with educational levels (5.6% to 2.8%, respectively). In women, the prevalence of current drinking categories (categories I and II) *increased* with educational level (45.1% to 73.3%, respectively). Regarding psychological distress, the prevalence of people presenting moderate or severe psychological distress *decreased* with educational levels in men (19.2% to 11.2%, respectively) and women (25.7% to 14.0%, respectively). Similar patterns were observed when income was used as an SES indicator, except for category III drinking in men, whose prevalence increased slightly with higher income levels (online supplemental table S2).

Deaths of despair mortality per 100 000 person-year was 29.4 (95% CI 27.2 to 31.7) and 62.6 (95% CI 59.1 to 66.2) in women and men, respectively. Lower education levels, higher psychological distress and increased alcohol consumption were associated with increased deaths of despair mortality in both men (table 1) and women (table 2). Severe psychological distress and being in the highest drinking category (men: cat. IV, women: cat. II) were each associated with more than a threefold increase in the risk of death of despair in both sexes (men: 3.65 (95% CI 2.97 to 4.49) and 3.55 (2.75–4.58), women: 3.12 (2.49–3.91) and 3.30 (2.46–4.42), respectively) compared with those with none/low distress or lifetime abstainers. Similar patterns were observed when SES was operationalised with income instead of educational level (online supplemental table S3).

**Table 1:** Hazard ratios of deaths of despair mortality, men

	Number of deaths	Sample size	HR	95% CI	P value
Education					
Bachelors (ref)	302	71 211	**1**	.	.
Some college	490	64 762	**1.46**	(**1.24 to 1.72**)	<0.001
Highschooll	951	106 490	**1.81**	(**1.55 to 2.13**)	<0.001
Alcohol use categories					
Lifetime abstainer (ref)	290	51 946	**1**	.	.
Former drinker	214	22 050	**1.50**	(**1.21 to 1.86**)	<0.001
Category I	799	138 954	0.97	(0.83 to 1.14)	0.707
Category II	191	18 062	**1.67**	(**1.35 to 2.07**)	<0.001
Category III	94	5964	**2.24**	(**1.71 to 2.93**)	<0.001
Category IV	155	5487	**3.55**	(**2.75 to 4.58**)	<0.001
Psychological distress					
None/low (ref)	1188	201 761	**1**	.	.
Moderate	386	33 769	**1.88**	(**1.64 to 2.16**)	<0.001
Severe	169	6933	**3.65**	(**2.97 to 4.49**)	<0.001
Race and ethnicity					
White participants (ref)	1254	162 417	**1**	.	.
Black participants	173	29 537	**0.73**	(**0.60 to 0.90**)	0.003
Hispanic participants	270	37 364	0.87	(0.73 to 1.03)	0.113
Others	46	13 145	**0.62**	(**0.43 to 0.88**)	0.007
Marital status					
Not married/cohabitating (ref)	955	95 648	**1**	.	.
Married/cohabiting	788	146 815	**0.55**	(**0.49 to 0.62**)	<0.001

Statistically significant results (p<0.05) are indicated in bold.

**Table 2:** Hazards ratios of deaths of despair mortality, women

	Number of deaths	Sample size	HR	95% CI	P value
Education					
Bachelors (ref)	144	81 417	**1**	.	.
Some college	334	90 233	**1.71**	(**1.30, 2.26**)	<0.001
Highschooll	593	139 858	**1.95**	(**1.49, 2.57**)	<0.001
Alcohol use categories					
Lifetime abstainer (ref)	348	119 326	**1**	.	.
Former drinker	102	17 633	**1.62**	(**1.25, 2.10**)	<0.001
Category I	525	164 667	1.12	(0.91, 1.36)	0.281
Category II	96	9882	**3.30**	(**2.46, 4.42**)	<0.001
Category III	.	.	.	.	.
Category IV	.	.	.	.	.
Psychological distress					
None/low (ref)	621	241 852	**1**	.	.
Moderate	306	56 098	**1.88**	(**1.59, 2.22**)	<0.001
Severe	144	13 558	**3.12**	(**2.49, 3.91**)	<0.001
Race and ethnicity					
White participants (ref)	748	200 342	**1**	.	.
Black participants	115	46 917	**0.65**	(**0.46, 0.91**)	0.012
Hispanic participants	166	48 731	**0.78**	(**0.61, 0.99**)	0.043
Others	42	15 518	0.94	(0.64, 1.37)	0.732
Marital status					
Not married/cohabitating (ref)	614	155 220	**1**	.	.
Married (ohabiting)	457	156 288	**0.61**	(**0.52, 0.71**)	<0.001

Statistically significant results (p<0.05) are indicated in bold.

Table 3 shows the association between educational level and deaths of despair mortality, decomposed into natural direct effect and indirect effect through psychological distress and alcohol use. The total effect of the low educational level compared with high educational level increased the hazard ratios of deaths of despair to 2.03 (95% CI 1.77 to 2.32) in men and 2.38 (95% CI 1.97 to 2.89) in women, and the total effect of medium educational level compared with high educational level increased the hazard ratios of deaths of despair to 1.66 (95% CI 1.43 to 1.93) in men and 1.91 (95% CI 1.54 to 2.36) in women. In men, up to 30% (low vs high education) of the total effect of the educational level on the deaths of despair was mediated through alcohol use and psychological distress, both contributing equally to the mediation between the educational level and deaths of despair mortality. In women, up to 20% of this effect was mediated through psychological distress, with no significant mediation by alcohol use. When income was operationalised to measure SES (online supplemental table S4), results were similar, except that psychological distress accounted for a larger share in the natural indirect effect in men (up to 24%) compared with alcohol use (6%) and that alcohol use had a negative mediating effect on deaths of despair in women.

**Table 3:** Effect of education on deaths of despair mortality decomposed into a direct effect and an indirect effect via alcohol use and psychological distress, by sex

	Men		Women	
	HR (95% CI)	% of TE (95% CI)	HR (95% CI)	% TE (95% CI)
Low education vs high education				
Total effect (TE) of low education	**2.03 (1.77, 2.32**)	**100**	**2.38 (1.97, 2.89**)	**100**
Natural direct effect of low education	**1.64 (1.42, 1.90**)	**70 (61, 77**)	**2.02 (1.65, 2.49**)	**81 (73, 88**)
Natural indirect effect of low education	**1.23 (1.19, 1.28**)	**30 (23, 38**)	**1.18 (1.12, 1.23**)	**19 (13, 27**)
Alcohol use: mediated	**1.10 (1.08, 1.13**)	**14 (10, 19**)	0.99 (0.96, 1.02)	−1 (−5, 2)
Psychological distress: mediated	**1.12 (1.1, 1.14**)	**16 (12, 22**)	**1.19 (1.15, 1.23**)	**20 (15, 28**)
Medium education vs high education				
TE of medium education	**1.66 (1.43, 1.93**)	**100**	**1.91 (1.54, 2.36**)	**100**
Natural direct effect of medium education	**1.47 (1.26, 1.71**)	**75 (63, 82**)	**1.72 (1.39, 2.13**)	**84 (74, 89**)
Natural indirect effect of medium education	**1.14 (1.11, 1.16**)	**25 (18, 36**)	**1.11 (1.09, 1.14**)	**16 (11, 25**)
Alcohol use: mediated	**1.06 (1.05, 1.07**)	**12 (8, 17**)	1.00 (0.99, 1.01)	0 (-2, 2)
Psychological distress: mediated	**1.07 (1.06, 1.08**)	**13 (9, 20**)	**1.11 (1.09, 1.13**)	**16 (11, 25**)

Statistically significant results (p<0.05) are indicated in bold.

Further decomposition analysis showed that the link between lower educational level and deaths of despair was primarily explained by higher exposure to psychological distress (in both sexes) and alcohol use (in men) rather than higher vulnerability to their effects. No evidence of differential vulnerability to alcohol use was found across educational groups. However, individuals with lower education showed less vulnerability to the effects of psychological distress (online supplemental table S5). When SES was measured by income, similar patterns were observed, though a negative differential vulnerability to alcohol use was observed in low- versus high-income groups (online supplemental table S6).

## Discussion

This study explored how SES, alcohol use and psychological distress contribute to deaths of despair in the USA, using mortality-linked nationally representative survey data. Severe psychological distress and being in the highest alcohol use category were each independently associated with over a threefold increased risk of death of despair in both men and women. Moreover, this study provides new evidence on distinct sex-specific contributions of alcohol use and psychological distress in the association between SES and deaths of despair. In men, both psychological distress and alcohol use mediated this association. In contrast, in women, only psychological distress mediated this association, but not alcohol use.

Our findings confirm that deaths of despair followed an increasing gradient from low to high SES, irrespective of whether SES was measured by education or income. Even when considering the mediating effect of alcohol use and psychological distress, SES remained a significant direct contributor to deaths of despair. This illustrates the complexity captured in the SES measure, which encompasses broader social determinants, such as early life conditions, social exclusion, living and working conditions, access to healthy food and transportation policy.^1 11^ These factors could also all contribute to the SES-deaths of despair association by different pathways than alcohol use or psychological distress. For instance, prior research has shown that counties with greater income inequality and limited social mobility have higher rates of deaths of despair.^33^

Psychological distress measures mental health vulnerability^25 26^ and captures dimensions of despair, such as feelings of hopelessness or worthlessness.^7^ People with lower SES reported higher levels of distress than people with higher SES, especially when SES was measured using income. This unequal distribution contributed substantially to the social gradient in deaths of despair: psychological distress explained up to 20% and up to 16% of the effect of low SES on deaths of despair in women and men, respectively. Chronic financial strain, housing instability, food insecurity and precarious employment are all stressors that accumulate disproportionately among low SES groups and contribute to mental health deterioration.^11^ This is particularly concerning in light of recent increasing distress and decreasing well-being for those of low SES.^34^

Alcohol use, particularly high to very high alcohol drinking categories, mediated 14% of the effect of low SES on deaths of despair in men. The association between SES and alcohol use is complex and seems to vary across sexes.^35^ Men with lower SES were more likely to be either abstinent (never or former drinkers) or in the high to very high drinking categories (>40–60 g/d to >60 g/day, respectively), while men with higher SES were more frequently found in the low to moderate drinking categories (>0–20 g/d to >20–40 g/d, respectively). The small share of men consuming over 40 g/d of alcohol, more frequently prevalent in those with low SES, seemed to have been a driver of social disparities in deaths of despair. This pattern is consistent with the alcohol harm paradox: individuals with lower SES experience disproportionately greater harm from alcohol consumption than their higher SES counterparts, despite similar or lower levels of use.^36^ This is consistent with previous studies showing that heavy episodic drinking could explain the higher alcohol-related mortality in lower SES.^37^

In contrast, this pattern was not observed in women. Women with lower SES were more often never or former drinkers, while women with higher SES were more frequently in the low to moderate drinking categories. The few women classified in the high to very high drinking categories were regrouped into the moderate drinking category for analysis, which may have diluted any potential mediating effect. This aligns with established evidence that men consume alcohol more heavily and more frequently than women.^13 38^ However, a convergence has also been observed over the last decades, raising concerns about a narrowing gender gap in heavy alcohol use.^39^ While men still die from deaths of despair twice as often as women, women’s deaths of despair have risen more quickly than men’s in recent years.^17^

To our knowledge, this is the first study to decompose the effect of SES on deaths of despair, identifying alcohol use and psychological distress as mediators using nationally representative US data. Its strengths include a large sample size, a long follow-up period and a rigorous analytical framework using marginal structural models and causal mediation analysis. Nevertheless, the following limitations need to be accounted for: First, we had no information on opioid use in the NHIS data, which contributes a large part to deaths of despair^5^ and disproportionately affects people with lower SES.^38^ Having this information might have allowed us to account for a larger part of the association between SES and deaths of despair. Second, we did not have a measure of heavy episodic drinking, which is particularly relevant for understanding alcohol-related harms in lower SES groups.^37^ Third, we did not have repeated measures of alcohol use and psychological distress over time, which could have led to a more nuanced understanding of the studied relationships. Fourth, these measures were self-reported, which may lead to recall and social desirability bias, resulting in an underestimation of their effect. Fifth, we were unable to capture the changes that occurred during the COVID-19 pandemic, particularly any potential increases in health disparities.^40 41^ Seventh, we could not account for additional confounders by unmeasured variables, such as adverse childhood experiences and family history of psychiatric or substance use disorders. This limitation restricts the causal interpretation and should be considered when interpreting the results. Finally, although the Kessler-6 scale is a validated scale for moderate and severe mental illness,^25 26^ we could not decompose despair into multiple dimensions as recommended by Shanahan *et al.*^7^ However, the four proposed dimensions of despair were captured with this scale (emotional, cognitive and biological) and alcohol use (behavioural).^7^

This study explored the pathways through which SES, psychological distress and alcohol use contribute to deaths of despair. While SES, psychological distress and alcohol use are major contributors to deaths of despair in both sexes, the pathways between SES and deaths of despair are sex-specific. Tailoring sex-specific prevention strategies and increasing access to interventions for people with low and medium SES might help curb the deaths of despair epidemic. Examining multiple dimensions of despair, as well as opioid use, and each cause of death of despair separately, could bring new perspectives on this complex phenomenon.^42^ Addressing broader social determinants remains a priority for improving health and requires concerted action by all sectors of society.^1 11^

## Supplementary material

10.1136/jech-2025-224372 online supplemental file 1

## Data Availability

Data may be obtained from a third party and are not publicly available.
