# Supplementary material for: Mediating role of psychological distress and alcohol use in socioeconomic disparities in deaths of despair: a causal mediation analysis using record linkage data
Source: J Epidemiol Community Health. 2025 Oct 9;80(1):e224372. doi: 10.1136/jech-2025-224372 (PMC12703257; doi:10.1136/jech-2025-224372)
Supplement: online supplemental file 1 [file jech-80-1-s001.pdf]

Supplemental material

Supplemental figure S1. Schematic diagram of the association between socioeconomic status (SES) and deaths of despair mortality, with mediators/moderators and covariates 2
Supplemental table S1. Description of the sample (N=553,971) by sex and educational level (unweighted Ns, weighted mean (SD) and weighted %). 3
Supplemental table S2. Description of the sample (N=553,971) by sex and income (unweighted Ns, weighted mean (SD) and weighted %) 4
Supplemental table S3. Hazards ratios of deaths of despair mortality, by sex, income as a measure of SES 5
Supplemental table S4. Effect of income on deaths of despair mortality decomposed into a direct effect and an indirect effect via alcohol use and psychological distress, by sex 6
Supplemental table S5. Effect of education on deaths of despair mortality decomposed into a natural direct effect, a natural indirect effect via alcohol use and psychological distress levels (differential exposure) and via the interaction between education and alcohol use and psychological distress (differential vulnerability), by sex 7
Supplemental table S6. Effect of income on deaths of despair decomposed into a natural direct effect, a natural indirect effect via alcohol use and psychological distress levels (differential exposure) and via the interaction between income and alcohol use and psychological distress (differential vulnerability), by sex. 8

Supplemental figure S1. Schematic diagram of the association between socioeconomic status (SES) and deaths of despair mortality, with mediators/moderators and covariates

A: natural direct effect of SES on deaths of despair
B: natural indirect effect of SES on deaths of despair, mediated by alcohol use and psychological distress (differential exposure)
B': natural indirect effect of SES on deaths of despair, moderated by alcohol use and psychological distress (differential vulnerability)
C: confounder effect

Supplemental table S1. Description of the sample (N=553,971) by sex and educational level (unweighted Ns, weighted mean (SD) and weighted %).

	**Overall**		**Men (N=242,463, 47.7%)**						**Women (N=311,508, 52.3%)**					
			**Highschool**		**Some college**		**Bachelors**		**Highschool**		**Some college**		**Bachelors**	
	N or mean (SD)	**%**	N or mean (SD)	**%**	N or mean (SD)	**%**	N or mean (SD)	**%**	N or mean (SD)	**%**	N or mean (SD)	**%**	N or mean (SD)	**%**
**N**	553971		106490		64762		71211		139858		90233		81417	
Years of follow-up	10.5 (6.2)		10.5 (6.2)		10.4 (6.2)		10.3 (6.2)		10.8 (6.2)		10.6 (6.2)		10.2 (6.2)	
Age	50.2 (16.1)		50.7 (16.3)		48.1 (15.1)		49.0 (15.1)		54.6 (17.4)		49.2 (15.7)		47.2 (14.7)	
Average alcohol use (g/day), among current drinkers	8.8 (22.7)		14.2 (36.0)		11.5 (22.4)		9.9 (22.0)		5.2 (17.4)		5.0 (11.5)		5.5 (11.1)	
**Alcohol use categories**														
Lifetime abstainer	171272	**28.8**	28095	**25.9**	11949	**18.3**	11902	**16.3**	71483	**48.7**	28542	**30.4**	19301	**22.9**
Former drinker	39683	**6.7**	12775	**11.1**	5598	**8.0**	3677	**4.8**	9203	**6.3**	5133	**5.4**	3297	**3.8**
Category I	303621	**57.2**	52294	**50.6**	38970	**61.6**	47690	**68.2**	55658	**42.4**	53420	**60.8**	55589	**69.3**
Category II	27944	**5.2**	7189	**6.7**	5048	**7.5**	5825	**8.0**	3514	**2.7**	3138	**3.5**	3230	**4.0**
Category III	5964	**1.1**	2867	**2.6**	1690	**2.5**	1407	**1.9**	.	**.**	.	**.**	.	**.**
Category IV	5487	**1.0**	3270	**3.0**	1507	**2.1**	710	**0.9**	.	**.**	.	**.**	.	**.**
**Psychological distress**														
None/low	443613	**81.2**	85622	**80.8**	53361	**83.0**	62778	**88.8**	102621	**74.3**	69767	**78.1**	69464	**86.0**
Moderate	89867	**15.5**	16521	**15.3**	9657	**14.5**	7591	**10.2**	28794	**20.0**	16629	**18.0**	10675	**12.6**
Severe	20491	**3.3**	4347	**3.9**	1744	**2.5**	842	**1.0**	8443	**5.7**	3837	**3.9**	1278	**1.4**
**Race and ethnicity**														
White participants	362759	**71.0**	62274	**64.1**	45870	**73.9**	54273	**78.6**	79329	**64.8**	61141	**73.4**	59872	**76.7**
Black participants	76454	**11.2**	15633	**12.4**	8492	**11.4**	5412	**6.7**	23420	**13.3**	14925	**13.3**	8572	**8.5**
Hispanic participants	86095	**12.6**	24941	**20.1**	7591	**10.5**	4832	**5.9**	31887	**17.8**	10765	**9.4**	6079	**6.1**
Others	28663	**5.3**	3642	**3.4**	2809	**4.1**	6694	**8.8**	5222	**4.1**	3402	**3.8**	6894	**8.8**
**Married/cohabitating**	303103	**68.0**	63127	**70.2**	37987	**70.9**	45701	**76.5**	64824	**59.8**	44885	**63.6**	46579	**70.7**

Supplemental table S2. Description of the sample (N=553,971) by sex and income (unweighted Ns, weighted mean (SD) and weighted %)

			Men (N=242,463, 47.7%)								Women (N=311,508, 52.3%)							
	Overall		Low		Medium		High		Missing		Low		Medium		High		Missing	
	N or mean (SD)	%	N or mean (SD)	%	N or mean (SD)	%	N or mean (SD)	%	N or mean (SD)	%	N or mean (SD)	%	N or mean (SD)	%	N or mean (SD)	%	N or mean (SD)	%
N	553971		56594		62076		82119		41674		94353		74026		82872		60257	
Years of followup	10.5 (6.2)		9.8 (6.0)		10.7 (6.2)		10.7 (6.2)		10.0 (6.2)		10.0 (6.1)		10.9 (6.2)		10.9 (6.3)		10.4 (6.2)	
Age	50.2 (16.1)		48.6 (16.7)		48.7 (16.2)		49.2 (14.0)		52.3 (16.6)		50.8 (18.1)		50.0 (16.6)		49.2 (13.9)		54.9 (17.4)	
Average alcohol use (g/day), among current drinkers	8.8 (22.7)		14.1 (38.9)		12.2 (29.3)		11.2 (19.6)		11.5 (32.5)		5.0 (18.3)		4.8 (11.5)		5.8 (13.1)		4.8 (11.6)	
**Alcohol use categories**																		
Lifetime abstainer	171272	**28.8**	16389	**29.3**	13413	**22.1**	11225	**13.5**	10919	**25.6**	47902	**49.9**	26302	**35.9**	17807	**21.3**	27315	**42.6**
Former drinker	39683	**6.7**	7306	**11.9**	5859	**8.9**	4838	**5.6**	4047	**8.9**	6813	**7.0**	3989	**5.3**	3251	**3.8**	3580	**5.6**
Category I	303621	**57.2**	26450	**48.0**	35060	**57.2**	54907	**67.8**	22537	**55.5**	37352	**40.6**	41487	**55.8**	57971	**70.3**	27857	**49.2**
Category II	27944	**5.2**	3338	**5.6**	4598	**7.0**	7578	**9.0**	2548	**6.2**	2286	**2.4**	2248	**3.0**	3843	**4.6**	1505	**2.5**
Category III	5964	**1.1**	1404	**2.3**	1591	**2.4**	2126	**2.5**	843	**2.0**	.	**.**	.	**.**	.	**.**	.	**.**
Category IV	5487	**1.0**	1707	**2.9**	1555	**2.4**	1445	**1.6**	780	**1.8**	.	**.**	.	**.**	.	**.**	.	**.**
**Psychological distress**																		
None/low	443613	**81.2**	41245	**73.3**	51820	**83.3**	72958	**88.9**	35738	**86.1**	63024	**66.9**	58664	**78.9**	71365	**86.1**	48799	**81.6**
Moderate	89867	**15.5**	11624	**20.3**	8881	**14.4**	8325	**10.1**	4939	**11.5**	23451	**24.9**	13069	**17.9**	10295	**12.4**	9283	**15.0**
Severe	20491	**3.3**	3725	**6.4**	1375	**2.3**	836	**1.0**	997	**2.4**	7878	**8.3**	2293	**3.2**	1212	**1.5**	2175	**3.3**
**Race/ethnicity**																		
White participants	362759	**71.0**	27854	**53.6**	42161	**71.3**	64425	**80.8**	27977	**71.0**	45735	**54.8**	50730	**72.8**	64739	**81.2**	39138	**70.7**
Black participants	76454	**11.2**	9971	**15.4**	7712	**10.9**	6718	**7.0**	5136	**10.8**	20737	**19.0**	10134	**11.3**	6942	**6.7**	9104	**12.3**
Hispanic participants	86095	**12.6**	15570	**25.2**	9369	**13.4**	6098	**6.6**	6327	**12.9**	23617	**21.1**	9820	**11.1**	6111	**6.0**	9183	**11.7**
Others	28663	**5.3**	3199	**5.8**	2834	**4.5**	4878	**5.5**	2234	**5.3**	4264	**5.1**	3342	**4.8**	5080	**6.1**	2832	**5.2**
**Married/cohabitating**	303103	**68.0**	27936	**62.1**	37044	**71.5**	55985	**78.6**	25850	**72.0**	30128	**44.7**	39057	**65.2**	57828	**79.4**	29275	**61.7**

Supplemental table S3. Hazards ratios of deaths of despair mortality, by sex, income as a measure of SES

	Men					Women				
	Number of deaths	Sample size	HR	95% CI	p-value	Number of deaths	Sample size	HR	95% CI	p-value
**Income**										
High (ref)	425	82119	**1**	**.**	.	178	82872	**1**	**.**	.
Medium	460	62076	**1.31**	**(1.12, 1.53)**	0.001	228	74026	**1.34**	**(1.01, 1.78)**	0.041
Low	579	56594	**1.86**	**(1.57, 2.22)**	<.001	511	94353	**2.27**	**(1.67, 3.07)**	<.001
Missing	279	41674	**1.32**	**(1.07, 1.62)**	0.009	154	60257	1.26	(0.92, 1.71)	0.145
**Alcohol use categories**										
Lifetime abstainer (ref)	290	51946	**1**	**.**	.	348	119326	**1**	**.**	.
Former drinker	214	22050	**1.52**	**(1.22, 1.89)**	<.001	102	17633	**1.59**	**(1.23, 2.07)**	<.001
Category I	799	138954	0.98	(0.83, 1.15)	0.82	525	164667	1.16	(0.94, 1.43)	0.178
Category II	191	18062	**1.73**	**(1.40, 2.15)**	<.001	96	9882	**3.47**	**(2.56, 4.69)**	<.001
Category III	94	5964	**2.36**	**(1.79, 3.09)**	<.001					
Category IV	155	5487	**3.76**	**(2.92, 4.85)**	<.001					
**Psychological distress**										
None/low (ref)	1188	201761	**1**	**.**	.	621	241852	**1**	**.**	.
Moderate	386	33769	**1.83**	**(1.59, 2.10)**	<.001	306	56098	**1.79**	**(1.52, 2.11)**	<.001
Severe	169	6933	**3.38**	**(2.73, 4.19)**	<.001	144	13558	**2.84**	**(2.26, 3.56)**	<.001
**Race/ethnicity**										
White participants (ref)	1254	162417	**1**	**.**	.	748	200342	**1**	**.**	.
Black participants	173	29537	**0.72**	**(0.59, 0.89)**	0.002	115	46917	**0.62**	**(0.44, 0.87)**	0.006
Hispanic participants	270	37364	**0.85**	**(0.72, 1.01)**	0.069	166	48731	**0.74**	**(0.59, 0.94)**	0.013
Others	46	13145	**0.55**	**(0.39, 0.79)**	0.001	42	15518	0.86	(0.59, 1.25)	0.434
**Marital status**										
Not married/cohabitating (ref)	955	95648	**1**	**.**	.	614	155220	**1**	**.**	.
Married/cohabitating	788	146815	**0.58**	**(0.51, 0.65)**	<.001	457	156288	**0.70**	**(0.59, 0.84)**	<.001

*Statistically significant results (p<0.05) are indicated in **bold***

Supplemental table S4. Effect of income on deaths of despair mortality decomposed into a direct effect and an indirect effect via alcohol use and psychological distress, by sex

	Men		Women	
	**HR (95% CI)**	**% of TE (95% CI)**	**HR (95% CI)**	**% TE (95% CI)**
**Low income vs. high income**				
Total effect (TE) of low income	**2.19 (1.92, 2.51)**	**100**	**3.03 (2.51, 3.65)**	**100**
Natural direct effect of low income	**1.74 (1.51, 2.01)**	**71 (62, 77)**	**2.50 (2.04, 3.07)**	**83 (76, 89)**
Natural indirect effect of low income	**1.26 (1.21, 1.32)**	**29 (23, 38)**	**1.21 (1.14, 1.29)**	**17 (11, 24)**
Alcohol use: mediated	**1.04 (1.02, 1.07)**	**6 (3, 9)**	0.96 (0.93, 1.00)	-3 (-7, 0)
Psychological distress: mediated	**1.21 (1.17, 1.25)**	**24 (19, 31)**	**1.25 (1.20, 1.31)**	**20 (15, 27)**
**Medium income vs. high income**				
TE of medium income	**1.44 (1.26, 1.65)**	**100**	**1.40 (1.13, 1.73)**	**100**
Natural direct effect of medium income	**1.33 (1.16, 1.54)**	**79 (64, 86)**	**1.34 (1.08, 1.67)**	**88 (60, 96)**
Natural indirect effect of medium income	**1.08 (1.06, 1.10)**	**21 (14, 36)**	**1.04 (1.02, 1.07)**	**12 (4, 36)**
Alcohol use: mediated	**1.02 (1.01, 1.03)**	**6 (2, 11)**	**0.97 (0.95, 0.99)**	**-10 (-27, -4)**
Psychological distress: mediated	**1.06 (1.05, 1.07)**	**15 (10, 26)**	**1.08 (1.06, 1.09)**	**22 (13, 62)**

*Statistically significant results (p<0.05) are indicated in **bold***

Supplemental table S5. Effect of education on deaths of despair mortality decomposed into a natural direct effect, a natural indirect effect via alcohol use and psychological distress levels (differential exposure) and via the interaction between education and alcohol use and psychological distress (differential vulnerability), by sex

	Men		Women	
	**HR (95% CI)**	**% of TE (95% CI)**	**HR (95% CI)**	**% TE (95% CI)**
**Low education vs. high education**				
Total effect (TE) of low education	**2.09 (1.83, 2.38)**	**100**	**2.49 (2.05, 3.01)**	**100**
Natural direct effect of low education	**1.76 (1.53, 2.02)**	**77 (69, 82)**	**2.30 (1.89, 2.80)**	**92 (85, 97)**
Natural indirect effect of low education	**1.19 (1.15, 1.23)**	**23 (18, 31)**	**1.08 (1.03, 1.14)**	**8 (3, 15)**
Alcohol use: differential exposure	**1.13 (1.06, 1.22)**	**17 (8, 28)**	1.03 (0.95, 1.12)	3 (-6, 13)
Alcohol use: differential vulnerability	0.95 (0.88, 1.02)	-7 (-18, 3)	0.94 (0.85, 1.02)	-7 (-18, 3)
Psychological distress: differential exposure	**1.18 (1.10, 1.27)**	**23 (14, 34)**	**1.33 (1.18, 1.50)**	**31 (18, 48)**
Psychological distress: differential vulnerability	**0.93 (0.87, 1.00)**	**-9 (-20, 0)**	**0.85 (0.75, 0.96)**	**-18 (-34, -5)**
**Medium education vs. high education**				
TE of medium education	**1.74 (1.51, 2.01)**	**100**	**2.07 (1.70, 2.53)**	**100**
Natural direct effect of medium education	**1.53 (1.32, 1.77)**	**76 (65, 83)**	**1.80 (1.47, 2.20)**	**80 (71, 87)**
Natural indirect effect of medium education	**1.14 (1.11, 1.18)**	**24 (17, 35)**	**1.15 (1.11, 1.20)**	**20 (13, 29)**
Alcohol use: differential exposure	**1.08 (1.04, 1.13)**	**14 (7, 24)**	1.02 (0.99, 1.06)	3 (-2, 8)
Alcohol use: differential vulnerability	0.99 (0.95, 1.04)	-2 (-11, 7)	0.99 (0.95, 1.03)	-2 (-7, 4)
Psychological distress: differential exposure	**1.10 (1.06, 1.14)**	**17 (10, 27)**	**1.19 (1.10, 1.28)**	**24 (13, 38)**
Psychological distress: differential vulnerability	0.97 (0.93, 1.01)	-6 (-14, 2)	0.96 (0.89, 1.04)	-5 (-18, 6)

Statistically significant results (p<0.05) are indicated in ***bold***

Supplemental table S6. Effect of income on deaths of despair decomposed into a natural direct effect, a natural indirect effect via alcohol use and psychological distress levels (differential exposure) and via the interaction between income and alcohol use and psychological distress (differential vulnerability), by sex.

	Men		Women	
	**HR (95% CI)**	**% of TE (95% CI)**	**HR (95% CI)**	**% TE (95% CI)**
**Low income vs. high income**				
Total effect of low Income	**2.21 (1.93, 2.53)**	**100**	**3.20 (2.66, 3.86)**	**100**
Natural direct effect of low income	**1.85 (1.59, 2.14)**	**77 (68, 85)**	**2.91 (2.39, 3.55)**	**92 (85, 98)**
Natural indirect effect of low income	**1.20 (1.13, 1.27)**	**23 (15, 32)**	**1.10 (1.03, 1.18)**	**8 (2, 15)**
Alcohol use: differential exposure	**1.09 (1.04, 1.15)**	**11 (4, 18)**	1.06 (0.95, 1.17)	5 (-4, 14)
Alcohol use: differential vulnerability	**0.91 (0.85, 0.97)**	**-13 (-21, -4)**	**0.87 (0.77, 0.98)**	**-12 (-22, -2)**
Psychological distress: differential exposure	**1.23 (1.13, 1.34)**	**26 (15, 39)**	**1.41 (1.23, 1.62)**	**30 (18, 43)**
Psychological distress: differential vulnerability	0.98 (0.89, 1.09)	-2 (-15, 11)	**0.85 (0.73, 0.98)**	**-14 (-28, -2)**
**Medium income vs. high income**				
Total effect of medium income	**1.49 (1.30, 1.70)**	**100**	**1.55 (1.26, 1.89)**	**100**
Natural direct effect of medium income	**1.37 (1.20, 1.58)**	**80 (66, 89)**	**1.45 (1.18, 1.79)**	**86 (67, 97)**
Natural indirect effect of medium income	**1.08 (1.05, 1.12)**	**20 (11, 34)**	**1.06 (1.01, 1.12)**	**14 (3, 33)**
Alcohol use: differential exposure	**1.05 (1.02, 1.08)**	**11 (4, 21)**	1.01 (0.96, 1.07)	3 (-11, 17)
Alcohol use: differential vulnerability	0.99 (0.95, 1.03)	-3 (-14, 7)	0.97 (0.91, 1.04)	-7 (-26, 9)
Psychological distress: differential exposure	**1.06 (1.03, 1.09)**	**15 (8, 25)**	**1.12 (1.07, 1.17)**	**26 (14, 51)**
Psychological distress: differential vulnerability	0.99 (0.96, 1.02)	-4 (-13, 4)	0.97 (0.91, 1.02)	-8 (-24, 5)

*Statistically significant results (p<0.05) are indicated in **bold***
